# Catechol-O-methyltransferase activity does not influence emotional processing in men

**DOI:** 10.1177/02698811221089032

**Published:** 2022-04-20

**Authors:** Marieke AG Martens, Nina Dalton, Jessica Scaife, Catherine J Harmer, Paul J Harrison, Elizabeth M Tunbridge

**Affiliations:** 1 Department of Psychiatry, University of Oxford, Oxford, UK; 2 Oxford Health NHS Foundation Trust, Oxford, UK

**Keywords:** Dopamine, tolcapone, mood, cognition, antidepressant, emotional processing

## Abstract

**Background:**

Catechol-O-methyltransferase (COMT) regulates cortical dopaminergic transmission and prefrontal-dependent cognitive function. However, its role in other cognitive processes, including emotional processing, is relatively unexplored. We therefore investigated the separate and interactive influences of COMT inhibition and Val^158^Met (rs4680) genotype on performance on an emotional test battery.

**Methods:**

We recruited 74 healthy men homozygous for the functional COMT Val^158^Met polymorphism. Volunteers were administered either a single 200 mg dose of the brain-penetrant COMT inhibitor tolcapone or placebo in a double-blind, randomised manner. Emotional processing was assessed using the emotional test battery, and mood was rated using visual analogue scales and the Profile of Mood States (POMS) questionnaire across the test day.

**Results:**

There were no main or interactive effects of Val^158^Met genotype or tolcapone on any of the emotional processing measures or mood ratings.

**Conclusions:**

Our findings suggest that, at least in healthy adult men, COMT has little or no effect on emotional processing or mood. These findings contrast with several neuroimaging studies that suggest that COMT modulates neural activity during emotional processing. Thus, further studies are required to understand how COMT impacts on the relationship between behavioural output and neural activity during emotional processing. Nevertheless, our data suggest that novel COMT inhibitors under development for treating cognitive dysfunction are unlikely to have acute off target effects on emotional behaviours.

## Introduction

Dopaminergic abnormalities are prominent in many psychiatric disorders and contribute substantially to their cognitive and emotional components (Figee et al., 2016; Goldman-Rakic et al., 2004). The catechol-O-methyltransferase (COMT) enzyme metabolises dopamine in the prefrontal cortex (PFC) (Byers et al., 2020; Tunbridge, 2004). The human *COMT* gene contains a functional polymorphism (Val^158^Met; rs4680) that influences enzyme activity: the ancestral Val^158^ allele has ~40% greater activity than the Met^158^ allele (Tunbridge et al., 2019). COMT activity can also be altered pharmacologically by COMT inhibitors, licenced for the adjunctive treatment of Parkinson’s disease (Zürcher et al., 1990).

An inverted-U-shaped relationship exists between dopamine and PFC-dependent cognitive function, whereby either too little or too much dopamine signalling impairs cognitive performance (Goldman-Rakic, 2000; Williams and Goldman-Rakic, 1995). Separately, both COMT inhibition (with the brain-penetrant COMT inhibitor tolcapone) and the Met^158^ allele are associated with improved PFC dopamine-dependent cognitive task performance, compared with placebo and the Val^158^ allele, respectively (Barkus et al., 2016; Byers et al., 2020; Farrell et al., 2012; Gasparini et al., 1997; Tan et al., 2007; Troudet et al., 2016; Tunbridge, 2004). Furthermore, and consistent with the inverted-U relationship, COMT genotype and tolcapone have interactive effects: COMT inhibition improves cognition in individuals with high COMT activity (e.g. Val^158^ homozygotes) but has less effect, or may even impair, cognition in those with low COMT activity (e.g. Met^158^ homozygotes) (Barkus et al., 2016; Farrell et al., 2012; Giakoumaki et al., 2008). Given these findings, COMT inhibitors with more optimal properties are under active development for the treatment of cognitive dysfunction in schizophrenia and other disorders (Byers et al., 2020; Su et al., 2021). However, COMT’s significance for clinically relevant domains other than executive cognition remains largely unexplored (DeBrosse et al., 2020; Korn et al., 2021; Tunbridge et al., 2012) and may have significance for understanding potential additional indications or side effects for these novel compounds.

Several lines of evidence suggest that COMT may influence emotional processing. Dopamine modulates brain regions implicated in the processing of emotional sensory information including the PFC, basolateral amygdala and nucleus accumbens (Gordon-Fennell and Stuber, 2021; Grace and Rosenkranz, 2002; Karreman and Moghaddam, 1996), and dopamine agonism is a potential therapeutic approach for the treatment of mood disorders (Dunlop and Nemeroff, 2007; Romeo et al., 2018). Evidence from both human and animal studies demonstrates that COMT genotype influences aspects of emotional processing. Neuroimaging studies demonstrate consistent associations between the COMT Val^158^Met polymorphism and brain activation (determined using fMRI) during emotional processing: the Met^158^ allele is associated with greater limbic brain activation (including hippocampus, amygdala and PFC) in response to negatively valanced stimuli (Drabant et al., 2006; Mier et al., 2010; Rasch et al., 2010; Smolka, 2005; Smolka et al., 2007; Williams et al., 2010; although see Kempton et al., 2009, who found greater limbic activation in Val^158^ vs Met^158^ homozygotes). Caution should be exercised when extrapolating simple differences in fMRI signal to underlying psychological processes; nevertheless, these findings are broadly consistent with a model in which the Met^158^ allele confers greater attention and/or sensitivity to negative emotional information, compared to the Val^158^ allele. This hypothesis is consistent with reported associations between the Met^158^ allele and risk for various anxiety and stress-related disorders (Domschke et al., 2004; Kolassa et al., 2010; Pooley et al., 2007). However, findings from behavioural studies are more mixed. Thus, while in some mouse models, lower COMT activity is associated with increased anxiety-like behaviours (Gogos et al., 1998; Papaleo et al., 2008), this is not seen for all COMT models (Barkus et al., 2016). Findings in human volunteers are also inconsistent: while some studies report exaggerated responses to negative stimuli and greater negative biases in processing emotional stimuli associated with the Met^158^, compared with the Val^158^, allele (Gohier et al., 2014; Gong et al., 2013; Montag et al., 2008), others have reported the opposite pattern (Weiss et al., 2007) or have found no genotype differences in emotional processing (Carrà et al., 2017; Defrancesco et al., 2011; Kempton et al., 2009).

To our knowledge, no studies have examined the impact of COMT inhibition on emotional processing in human participants or whether its effects interact with genotype (and, if so, whether this also follows an inverted-U). However, in our earlier study, we found that tolcapone increased visual analogue score ratings of ‘happiness’ in Val^158^ but not Met^158^ individuals (Farrell et al., 2012). Further hints of a potential impact of COMT inhibition on emotional processing come from a small, open clinical trial that provided preliminary evidence that tolcapone might have antidepressant potential (Fava et al., 1999).

Taken together, the available data suggest that COMT genotype influences limbic activation during emotional processing; however, effects on emotional behaviour are not clear-cut. Furthermore, although there are hints that COMT inhibition may positively impact on mood, these findings are preliminary and in need of replication. It is also not known if or how these effects differ based on COMT genotype. This study, therefore, aimed to comprehensively assess the impact of COMT genotype and COMT inhibition on emotional processing. To achieve this, we investigated the separate and interactive effects of COMT inhibition and Val^158^Met genotype on emotional processing in healthy men, using an emotional test battery which tests multiple dimensions of emotional behaviours and is sensitive to antidepressant actions (Harmer et al., 2011).

## Methods

We performed a double-blind, randomised, placebo-controlled, experimental medicine study that investigated non-smoking healthy males homozygous for the COMT Val^158^Met polymorphism. The study’s design paralleled that of our study of tolcapone’s effects on working memory (Farrell et al., 2012).

### Participants

Ethical approval for this study was granted by the South Central – Berkshire Research Ethics Committee (REC number: 14 SC 0035). All participants gave written informed consent.

Non-smoking, healthy men aged 18–45 years old were recruited by advertisement. Women were excluded from the study given marked sexually dimorphic effects of COMT (Harrison and Tunbridge, 2008; Laatikainen et al., 2013). To identify homozygotes, males who expressed initial interest in the study were mailed study packs and provided buccal swabs by post. All returned samples were genotyped for the COMT Val^158^Met polymorphism (rs4680) using the appropriate Taqman SNP Genotyping Assay.

Potential participants (homozygotes), who were still interested in taking part, underwent telephone screening to establish inclusion and exclusion criteria. Mental health was assessed using the Structured Clinical Interview for *DSM-IV* (*Diagnostic and Statistical Manual of Mental Disorders*). Exclusion criteria were a current or past history of psychiatric or neurological disorder, use of psychotropic medication, alcohol intake greater than 30 units/week, illicit drug use in the last 3 months or an inadequate command of spoken English. Due to the small risk of hepatotoxicity accompanying tolcapone use, participants were also questioned about their medical history and whether they had any history of liver disease. The participants’ general practitioners were asked to check that participants did not meet any of the exclusion criteria and that there were no medical reasons why it would be unsafe for them to take part in the study.

In all, 369 males were genotyped; genotypes were in Hardy–Weinberg equilibrium (*n* = 90 Val^158^/Val^158^; *n* = 198 Val^158^/Met^158^ and *n* = 81 Met^158^/Met^158^; χ^2^ = 2.01; *p* = 0.156). Not all genotyped homozygotes accepted the subsequent screening invitation, and not all proved eligible after completion of screening. A total of 74 men were recruited, to match the sample size of our previous study (Farrell et al., 2012), and randomised (by P.J.H.) to receive either placebo or tolcapone. The participants and the main study researchers (J.S., N.D. and M.A.G.M.) were blind to both genotype and drug allocation.

### Study design

Participants attended the laboratory for testing at 9.15 a.m. Upon arrival, they were reminded of the study details and their right to withdraw. Once consent had been reaffirmed, participants were asked to complete the National Adult Reading Test (NART – a measure of verbal IQ), along with the State-Trait Anxiety Inventory (STAI) (Spielberger, 1983). Furthermore, baseline measures of mood were taken by using the Profile of Mood States (POMS) questionnaire (McNair et al., 1971) and visual analogue scales (VAS – rating alertness, drowsiness, anxiety, sadness, happiness and nausea). After participants had completed these initial paper measures, they were given a tolcapone tablet (200 mg) or visually matched placebo by mouth. Tolcapone has an elimination half-life of 2.0 ± 0.8 h; the dose given produces 70–80% peripheral blood COMT inhibition between 1 and 4 h (Ceravolo et al., 2002; Dingemanse et al., 1995) and influences cognitive function within this period (Tunbridge et al., 2012). Therefore, testing commenced 90 min after administration and was completed within 4 h.

Participants completed the emotional test battery (Harmer et al., 2004), a behavioural test battery that has previously shown to be sensitive to detect biases in emotional processing. The POMS and VAS were repeated three more times during the test day: at 1.5 h and 3.5 h after tablet administration and once testing was completed. However, due to experimenter error, data for the 1.5 h and 3.5 h timepoints were incomplete, and so, only data from the pre- and post-testing timepoints are presented here.

### Tasks

The emotional test battery comprised four validated, computerised cognitive tasks designed to assess the processing of a variety of emotionally valanced stimuli. It is sensitive to the negative biases in emotional processing observed in depression and to the early effects of antidepressants on emotional processing (Harmer et al., 2009). The order of administration was as follows: Facial Expression Recognition Task (FERT), Emotional Categorisation Task (ECAT), Faces Dot Probe Task (FDOT) and Emotional Recall Task (EREC).

#### Facial Expression Recognition Task (FERT)

Participants were presented with pictures of human facial expressions. Each face displayed one of six basic emotions (anger, disgust, fear, happiness, sadness or surprise). Each emotional expression was presented at different levels of intensity (10%, 20%, 30%, 40%, 50%, 60%, 70%, 80%, 90% and 100%), which have been created by combining shape and texture features of the two extremes ‘neutral’ (i.e. 0%) and ‘full prototypical emotion’ (i.e. 100%) to varying degrees (Young et al., 1997). In total, four examples of each emotion at each intensity level were presented. Emotions were displayed by 10 different individuals overall, and for each of the 10 individuals, a neutral facial expression was presented as well. Thus, 250 stimulus presentations (6 emotions × 10 intensities × 4 examples + 10 neutral faces) were used in total. Facial expressions were presented in a random order on a computer screen for approximately 500 ms, followed by a blank black screen. Participants were instructed to correctly classify each facial expression as quickly and as accurately as possible. Responses were made by pushing one out of seven labelled buttons on a keyboard. The main outcomes of interest were accuracy, misclassifications and averaged reaction time for correct classifications.

#### Emotional Categorisation Task (ECAT)

The ECAT comprises a series of positively and negatively valanced self-referent words, and participants were required to indicate whether they would like or dislike to be referred to as each word. In total, 40 words describing either extremely agreeable/positive characteristics (e.g. ‘cheerful’, ‘honest’, ‘optimistic’) or extremely disagreeable/negative characteristics (e.g. ‘domineering’, ‘untidy’, ‘hostile’) were presented individually in the centre of the screen for approximately 500 ms each. Positive and negative words were chosen to be comparable with regard to frequency, length and meaningfulness and were presented in a random order. Responses were made by pressing correspondingly labelled buttons on a keyboard. The main outcome of interest was the averaged reaction time for correct classifications, but accuracy was recorded to make sure participants were engaged with the task.

#### Faces Dot Probe Task (FDOT)

In the FDOT, two faces (either an emotional (fearful or happy) or a neutral face) were shown at the top and at the bottom of the computer screen and then replaced by a pair of dots to which the participant had to respond by indicating whether the dots are vertically or horizontally aligned by pressing a labelled key on the keyboard. On half of the total 192 trials, the faces were presented very briefly and immediately switched to a muddled face mask. Attentional vigilance scores were calculated by subtracting the mean reaction time from trials when probes appeared in the same position as the emotional face (congruent trials) from trials when probes appeared in the opposite position to the emotional face (incongruent trials).

#### Emotional Recall Task (EREC)

The EREC is a surprise free recall task during which participants were required to remember as many of the positively and negatively valanced self-referent words from the ECAT as they could in 4 min. The main outcomes of interest were the numbers of correctly and incorrectly recalled words.

### Data analysis

Analyses were carried out using SPSS for Windows (Version 25, IBM SPSS Statistics).

Due to the non-normality of the raw data, we calculated change scores for VAS and POMS measures (baseline to post-testing). These were analysed by univariate analysis of variance (ANOVA) with COMT genotype (Met/Met vs Val/Val) and drug (placebo vs tolcapone) as between-subjects factors.

Emotional processing measures were analysed using repeated-measures ANOVA with COMT genotype (Met/Met vs Val/Val) and drug (placebo vs tolcapone) as between-subjects factors and emotion/valence as the within-subjects factor. Additional within-subjects factors were included for the FDOT (mask condition) and EREC (recall accuracy). Huynh–Feldt correction was applied where data failed Mauchly’s Test of Sphericity. Planned post hoc comparisons were used to explore statistically significant interaction terms.

## Results

The demographics of the final sample are shown in Table 1. There were no robust effects of Val^158^Met genotype, inhibition or their interaction on any of the emotional processing measures (Table 2). Thus, while there were main effects of emotion on accuracy, misclassifications and reaction time (*F*s > 21; *p*s < 4.7 × 10^–37^) during facial emotion recognition, there were no main or interactive effects of genotype or drug (*F*s < 1.22; *p*s > 0.31). There was a main effect of valence on reaction time (*F*_1,69_ = 32.0; *p* = 3.2 × 10^–7^) but not accuracy (*F*_1,69_ = 0.9; *p* = 0.35) during assessment of word valence but no other main or interactive effects (*F*s < 0.61; *p*s > 0.05); similar findings were obtained for their later recall (main effect of valence for correct recalls: *F*_1,69_ = 12.9; *p* = 0.001; main effect of valence for errors: *F*_1,69_ = 6.0; *p* = 0.017; other effects: *F*s < 0.55; *p*s > 0.47). Finally, other than a main effect of emotion (*F*_1,68_ = 11.6; *p* = 0.001), there were no other main or interactive effects on attentional vigilance towards happy or fearful faces (*F*s < 1.61; *p*s > 0.12).

**Table 1.** Demographics of participants.

	Met Placebo	Val Placebo	Met Tolcapone	Val Tolcapone
**Number**	19	19	19	17
**Age (years)**	23.3 ± 1.0	25.3 ± 1.3	24.8 ± 1.0	22.5 ± 1.2
**NART predicted Full Scale IQ** ^a^	117.1 ± 1.2	115.6 ± 1.8	115.9 ± 1.5	113.9 ± 1.7
**Baseline State Anxiety** ^b^	28.2 ± 1.2	28.1 ± 1.2	31.4 ± 1.8	30.2 ± 1.1
**Baseline Trait Anxiety** ^b^	30.6 ± 1.7	32.1 ± 1.4	34.4 ± 2.1	32.1 ± 1.4

NART: National Adult Reading Test.

Values are mean ± SEM.

a Non-native speakers excluded.

b Spielberger’s State-Trait Anxiety Inventory.

**Table 2.** Behavioural measures of emotional processing measured using the Emotional Test Battery (ETB).

	Val Placebo	Met Placebo	Val Tolcapone	Met Tolcapone
**Facial** **Expression** **Recognition** **Task (FERT)**				
**Accuracy (%)**				
**Anger**	46.3 ± 2.3	50.0 ± 2.1	51.9 ± 3.0	46.7 ± 3.2
**Disgust**	42.5 ± 3.3	47.9 ± 3.0	44.2 ± 4.1	47.0 ± 4.0
**Fear**	43.3 ± 3.4	44.6 ± 4.1	45.2 ± 3.5	48.8 ± 3.6
**Happy**	68.5 ± 2.6	66.9 ± 2.0	63.1 ± 1.8	65.0 ± 3.1
**Sad**	44.6 ± 2.6	49.6 ± 2.3	50.0 ± 4.0	50.8 ± 3.6
**Surprise**	57.6 ± 2.0	55.7 ± 2.8	58.0 ± 2.1	54.5 ± 2.7
**Neutral**	68.9 ± 4.5	69.4 ± 4.3	71.9 ± 3.9	71.1 ± 3.8
**Misclassifications (%)**				
**Anger**	14.5 ± 2.2	14.1 ± 1.7	13.1 ± 1.6	14.2 ± 1.2
**Disgust**	10.4 ± 2.0	7.3 ± 1.5	7.5 ± 1.5	9.0 ± 1.4
**Fear**	8.0 ± 1.3	8.2 ± 1.4	7.1 ± 1.1	7.1 ± 0.9
**Happy**	2.2 ± 0.6	3.4 ± 0.9	2.8 ± 0.6	5.5 ± 2.1
**Sad**	8.79 ± 1.4	9.94 ± 1.9	8.31 ± 2.2	8.89 ± 1.7
**Surprise**	10.31 ± 1.4	12.83 ± 1.8	11.00 ± 2.0	11.94 ± 1.6
**Neutral**	63.26 ± 4.1	67.33 ± 4.7	67.75 ± 4.6	64.89 ± 3.3
**Reaction time** (**ms**)				
**Anger**	1798 ± 91	1820 ± 107	1795 ± 81	1776 ± 84
**Disgust**	1794 ± 120	1897 ± 113	1800 ± 67	2009 ± 113
**Fear**	2122 ± 75	2167 ± 117	2104 ± 144	2104 ± 107
**Happy**	1653 ± 64	1603 ± 53	1598 ± 51	1604 ± 74
**Sad**	1858 ± 71	1973 ± 98	1907 ± 87	1866 ± 91
**Surprise**	1907 ± 122	1822 ± 95	1817 ± 72	1951 ± 98
**Neutral**	1748 ± 126	1725 ± 149	1655 ± 116	1520 ± 92
**Emotional** **Categorisation** **Task (ECAT)**				
**Reaction time (ms)**				
**Positive**	1047 ± 64	1104 ± 50	1132 ± 42	1073 ± 51
**Negative**	1158 ± 59	1122 ± 37	1224 ± 54	1163 ± 58
**Faces** **Dot** **Probe** **Task (FDOT)**				
**Vigilance bias score (ms)**				
**Unmasked positive**	11 ± 9	−18 ± 8	−4 ± 9	−11 ± 7
**Unmasked negative**	6 ± 9	−4 ± 9	−1 ± 7	20 ± 11
**Masked positive**	−10 ± 10	−4 ± 11	−15 ± 7	−24 ± 10
**Masked negative**	10 ± 11	6 ± 10	9 ± 9	10 ± 7
**Emotional** **Recall** **Task (EREC)**				
**Hits (n correct)**				
**Positive**	4.0 ± 0.5	4.9 ± 0.6	4.0 ± 0.6	4.8 ± 0.5
**Negative**	3.1 ± 0.5	3.9 ± 0.4	3.5 ± 0.5	3.6 ± 0.5
**Intrusions (n wrong)**				
**Positive**	2.2 ± 0.5	1.8 ± 0.4	1.7 ± 0.3	2.1 ± 0.4
**Negative**	1.7 ± 0.6	0.9 ± 0.3	1.3 ± 0.3	1.2 ± 0.6

Values are mean ± SEM.

**Figure 1.**
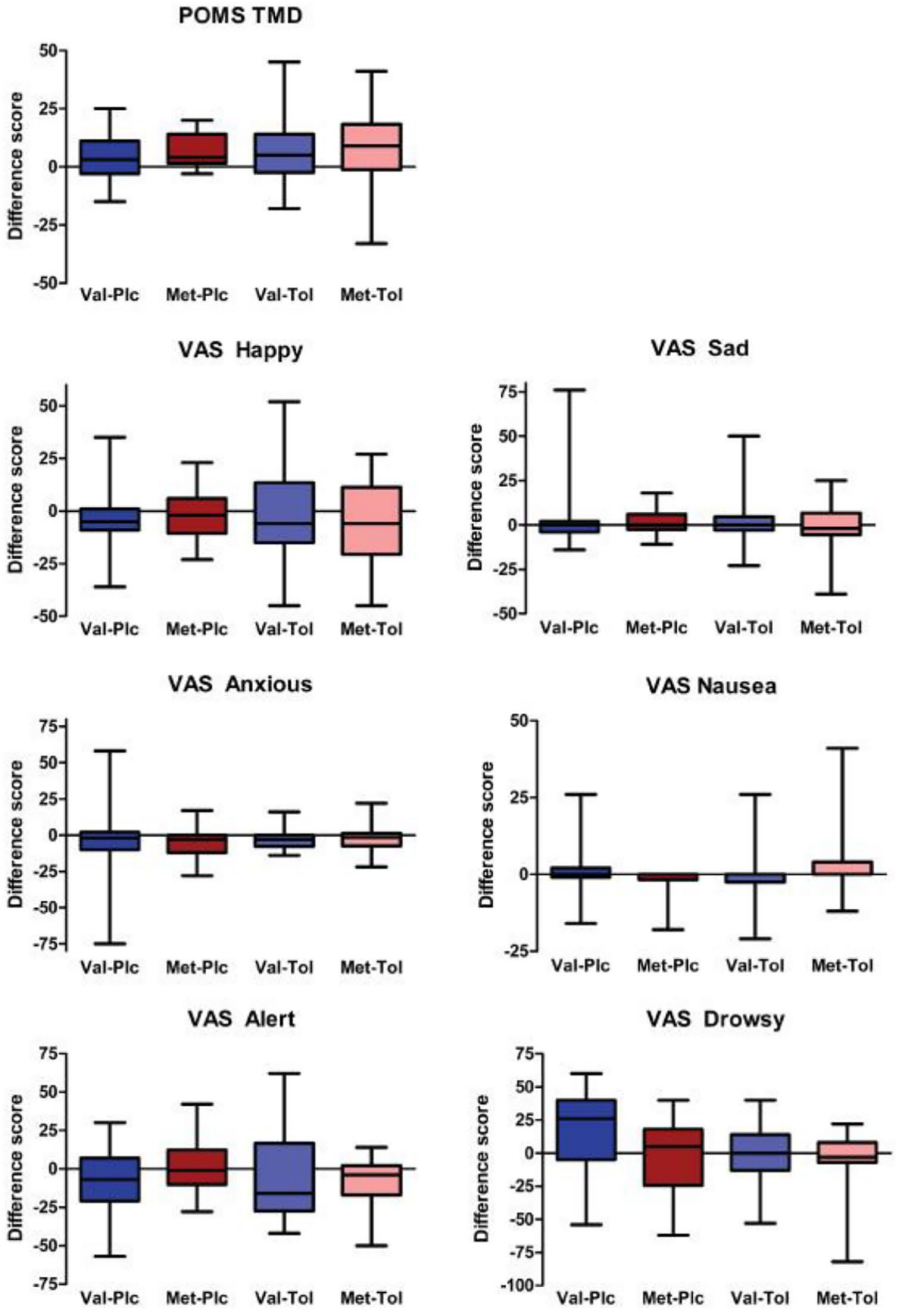
Changes in subjective ratings in the different genotype and drug groups. The change scores from pre–post drug timepoints are shown broken down by group. Boxes indicate the median, 25th and 75th percentile values, with the whiskers indicating the maximum and minimum values. Only self-rated drowsiness and nausea differed between groups (see text for details). POMS TMD: Profile of Mood States Total Mood Disturbance; VAS: visual analogue scales; Plc: Placebo; Tol: Tolcapone.

Comparing pre- and post-testing timepoints, there were no main or interactive effects on change scores for subjective mood VAS ratings (i.e. happiness, sadness or anxiety: *F*s < 0.43; *p*s > 0.51) or total mood disturbance (POMS) (*F*s < 0.39; *p*s > 0.54; Figure 1). There was a main effect of drug on VAS drowsiness (*F*_1,69_ = 4.2; *p* = 0.044; change in drowsiness greater in tolcapone than placebo) in the absence of other main or interactive effects (*F*s < 3.0; *p*s > 0.089; Figure 1). However, there were no effects on VAS alertness ratings (*F*s < 1.4; *p*s > 0.24; Figure 1). Finally, there was a drug × genotype interaction for VAS nausea ratings (*F*_1,69_ = 4.4; *p* = 0.039) in the absence of other main effects (*F*s < 1.1; *p*s > 0.31), due to a genotype difference (Val > Met; *p* = 0.03) in the placebo group that was not observed in the tolcapone group (*p* = 0.44).

## Discussion

We found no effect of either COMT inhibition or COMT Val^158^Met genotype on performance on an emotional test battery or any effects on subjective mood ratings. Thus, our findings suggest that, at least in healthy men, COMT does not influence behavioural measures of emotional processing. To our knowledge, ours is the first study to examine the effect on COMT genotype on multiple aspects of emotional processing and mood within a single cohort of human volunteers as well as the first to assess the impact of acute COMT inhibition on these processes. Given the similarities in experimental design and study population, the current data suggest that our previous finding of a positive effect of tolcapone on subjective happiness ratings represents a false positive (Farrell et al., 2012), consistent with a lack of effect of COMT genotype on subjective mood ratings reported elsewhere (Gibbs et al., 2014).

Detection of facial emotions is the area of emotional behaviour that is best studied with respect to COMT genotype, although a variety of different experimental approaches have been employed. Our findings agree with most earlier studies showing no overall effect of COMT genotype on facial emotion detection (Carrà et al., 2017; Defrancesco et al., 2011; Kempton et al., 2009; Soeiro-de-Souza et al., 2012; Tamm et al., 2016). We cannot rule out more subtle effects of COMT on detection of specific types of emotional information. However, it is notable that where more subtle differences have been found, results are inconsistent. For example, while some studies found genotype effects that were specific to negative emotions (Lischke et al., 2019; Weiss et al., 2007), other studies report altered detection of neutral, but not emotional, facial expressions associated with COMT (Erkoreka et al., 2020; Gohier et al., 2014).

Our negative behavioural findings are of interest in the context of the better-studied relationship between COMT genotype and brain activation during emotional processing. Thus, although there are some conflicting reports (Bishop et al., 2006; Domschke et al., 2012), most studies (including by meta-analysis) suggest that the Met allele is associated with greater limbic activation during emotional processing (Drabant et al., 2006; Mier et al., 2010). There is also some evidence that COMT genotype influences patterns of functional connectivity during emotional processing (Opmeer et al., 2013; Vai et al., 2017; but see Surguladze et al., 2012). Notably, this disconnect between neural measures and behavioural effects has been observed within subjects, both in the specific case of COMT genotype (Kempton et al., 2009) and after other manipulations of dopamine function (Martens et al., 2021). It is possible that that measurements of quantitative biological traits, such as BOLD response, are more sensitive to the subtle effects of genetic variation than studies looking at overt behaviour (Meyer-Lindenberg and Weinberger, 2006). Alternatively, in the case of genetically encoded differences such as COMT Val^158^Met that presumably affect enzyme activity throughout development (Tunbridge et al., 2007), it is possible that differences observed at the neural level represent compensatory changes in the precise circuitry used to achieve a given behavioural outcome. It may be that such subtle differences in ‘wiring’ are insufficient to lead to any observable behavioural differences in healthy controls under baseline conditions. It is therefore notable that there is some evidence, albeit inconsistent, for associations between COMT genotype and emotion-related behaviours that are modulated by environmental adversity (Asselmann et al., 2018; Evans et al., 2009; Lovallo et al., 2017). Given the difficulty in disentangling such effects in epidemiological studies, it will be of interest to investigate the impact of stressors and other challenges on the relationships between COMT activity and emotional processing under controlled laboratory conditions.

Taken together, our data suggest that variation in COMT activity, whether genetic or pharmacological in origin, has no demonstrable effect on mood and emotional processing in healthy men under baseline conditions. Nevertheless, given the design and demographics of this study, further research is required to investigate the extent to which our findings generalise to other populations, to establish how COMT-related differences in neural processing relate to emotional behaviours and to assess whether prolonged COMT inhibition affects emotional processing. Our findings do not have immediate therapeutic relevance, since the licenced COMT inhibitors are either not brain-penetrant or are limited by hepatotoxicity. However, our results suggest that the novel, brain-penetrant COMT inhibitors under development for cognitive dysfunction (Byers et al., 2020) are unlikely to have major off-target effects on mood and related behaviours, at least when administered acutely.
